# Atlas-based analysis of cardiac shape and function: correction of regional shape bias due to imaging protocol for population studies

**DOI:** 10.1186/1532-429X-15-80

**Published:** 2013-09-13

**Authors:** Pau Medrano-Gracia, Brett R Cowan, David A Bluemke, J Paul Finn, Alan H Kadish, Daniel C Lee, Joao AC Lima, Avan Suinesiaputra, Alistair A Young

**Affiliations:** 1 Department of Anatomy with Radiology, Faculty of Medical and Health Sciences, University of Auckland, 85 Park Road, Auckland 1142, New Zealand; 2 National Institute of Biomedical Imaging and Bioengineering, Bethesda, Maryland, USA; 3 Department of Radiology, UCLA, Los Angeles, USA; 4 Feinberg Cardiovascular Research Institute, Northwestern University, Chicago, USA; 5 The Donald W. Reynolds Cardiovascular Clinical Research Center, The Johns Hopkins University, Baltimore, USA

**Keywords:** Cardiovascular magnetic resonance, Atlas, Bias correction

## Abstract

**Background:**

Cardiovascular imaging studies generate a wealth of data which is typically used only for individual study endpoints. By pooling data from multiple sources, quantitative comparisons can be made of regional wall motion abnormalities between different cohorts, enabling reuse of valuable data. Atlas-based analysis provides precise quantification of shape and motion differences between disease groups and normal subjects. However, subtle shape differences may arise due to differences in imaging protocol between studies.

**Methods:**

A mathematical model describing regional wall motion and shape was used to establish a coordinate system registered to the cardiac anatomy. The atlas was applied to data contributed to the Cardiac Atlas Project from two independent studies which used different imaging protocols: steady state free precession (SSFP) and gradient recalled echo (GRE) cardiovascular magnetic resonance (CMR). Shape bias due to imaging protocol was corrected using an atlas-based transformation which was generated from a set of 46 volunteers who were imaged with both protocols.

**Results:**

Shape bias between GRE and SSFP was regionally variable, and was effectively removed using the atlas-based transformation. Global mass and volume bias was also corrected by this method. Regional shape differences between cohorts were more statistically significant after removing regional artifacts due to imaging protocol bias.

**Conclusions:**

Bias arising from imaging protocol can be both global and regional in nature, and is effectively corrected using an atlas-based transformation, enabling direct comparison of regional wall motion abnormalities between cohorts acquired in separate studies.

## Background

Cardiovascular imaging studies are becoming increasingly common, both to determine surrogate endpoints in clinical trials [1] and to investigate epidemiological manifestations of cardiac disease [2,3]. Although great effort and expense is usually expended on obtaining excellent quality cardiovascular imaging data, the images are typically used only for study-specific outcomes, and are unavailable for wider use. By pooling image data across multiple studies, valuable data can be re-used and combined in novel ways. In the brain, imaging studies have been used extensively in combination with atlas-based analysis methods in order to demonstrate morphological changes due to disease [4,5]. Computational, structural and functional atlases can be generated which map related scientific information to spatial coordinates. Recently, these methods have begun to be applied to the analysis of cardiac shape and motion [6]. For example, Lewandowski *et al.* used an atlas-based analysis to characterize clinically important shape changes between individuals born preterm and term-born controls [7]. These methods provide novel quantitative information which can be obtained retrospectively and applied across multiple studies for comparisons between populations.

However, application of these methods to multiple studies with different imaging protocols is problematic due to the variety of cardiovascular imaging modalities and methods employed, and the lack of tools with which data can be meaningfully pooled into large multi-study meta-analyses. Requiring all studies to use a common imaging protocol is too restrictive, therefore *a posteriori* corrections must be made to quantify and remove these sources of bias. If corrections could be made on a regional basis which account for protocol bias, data from clinical studies obtained using different methodologies or even modalities could be compared or combined.

The Cardiac Atlas Project is a worldwide consortium seeking to pool cardiac imaging data in a standardized manner from multiple studies in order to facilitate meta-analyses [8]. Data are de-identified in a HIPAA compliant manner, annotated using standard ontological schema, stored in a web-accessible picture archiving and communication system (PACS) database, and analyzed using atlas-based techniques [8]. Approximately 3000 cardiovascular magnetic resonance (CMR) cases have been contributed to date and the data are available on request from the website. Two major studies which have contributed data to the CAP database are:

i. the Multi-Ethnic Study of Atherosclerosis (MESA) [2], comprising asymptomatic volunteers imaged using gradient recalled echo (GRE), and

ii. the Defibrillators to Reduce Risk by Magnetic Resonance Imaging Evaluation (DETERMINE) study [9], comprising patients with a history of myocardial infarction imaged using steady state free precession (SSFP).

Comparison of statistical shape differences between these two cohorts is clinically interesting because precise shape differences between sub-clinical and clinical populations could be quantified. However, such comparisons require compensation of any bias arising due to the different imaging protocols: in this case GRE and SSFP.

It is well known that, globally, SSFP gives rise to larger estimates of left ventricle (LV) cavity volume and smaller estimates of LV mass than GRE [10]. However, the regional effects of these two imaging protocols on statistical shape representations of the heart are unknown. Image contrast between blood and myocardium in GRE images is highly influenced by local blood in-flow effects, whereas SSFP images have reduced dependence on flow due to the intrinsic T1/T2 contrast. We therefore hypothesized that regional differences in shape may be identified between GRE and SSFP imaging protocols.

In this paper, we propose an atlas-based method for the correction of shape bias arising from imaging protocol. We investigate whether both regional and global bias can be corrected, and whether this correction can improve the detection of statistical differences in regional shape and motion between cohorts.

## Methods

### Transformation from GRE to SSFP

**Figure 1:**
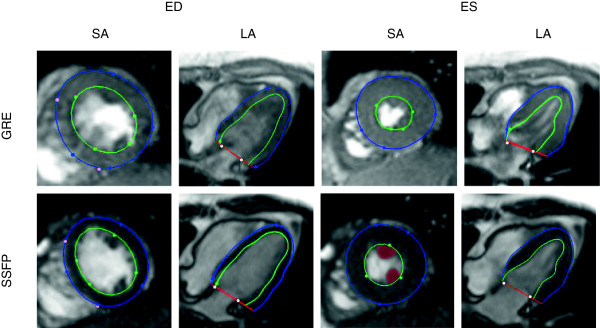
**Image and shape differences for a volunteer imaged both with GRE (top), and SSFP (bottom), for the same short-axis (SA), long-axis (LA) planes at end diastole (ED) and end systole (ES).** Green and blue contours and markers show the model’s endocardial and epicardial boundaries and guide points, respectively. Light color markers denote fiducial landmarks (right ventricular free wall insertion points, mitral valve hinge points) used to define the location of the model shape parameters in consistent positions relative to the anatomy of the heart. Papillary muscles are highlighted in red in the SSFP SA slice at ES.

In order to correct for the effects of GRE vs. SSFP imaging, we generated a transformation which corrected for bias on a local level (Figure 1). The aim of this shape bias correction is to remove systematic differences in shape over a population, so that on average the GRE-derived heart shapes appear to have been produced by SSFP imaging.

To generate the transformation, 46 healthy volunteers (26 males aged 42.5 ± 11.7 years and 20 females aged 37.3 ± 13.9 years) were scanned with both GRE and SSFP protocols on a Siemens 1.5 T scanner (Siemens Medical Solutions, Erlangen, Germany). GRE imaging parameters were: echo time 3.54 ms, repetition time 66.87 ms, flip angle 20°, matrix size 256×144, slice thickness 6 mm (0 mm gap between adjacent slices), flow compensation, and FOV 360×360 cm. SSFP imaging parameters were: echo time 1.41 ms, repetition time 60.66 ms, flip angle 77°, matrix size 256×144, slice thickness 6 mm (0 mm gap between adjacent slices), and FOV 360×360 cm.

### The shape atlas

Guide-point modeling [11] was used to interactively customize a time-varying 3D finite element model of the LV to fit each subject’s images using custom software (CIM version 6.0, University of Auckland, New Zealand). The model comprised 16 bicubic finite elements with *C*^*1*^ continuity, defined in a prolate spheroidal coordinate system. This enabled an efficient representation of the shape of the left ventricle as a radial function of two angular coordinates, with only 215 parameters (see [11,12] for details). Briefly, the model was interactively fitted by least-squares optimization to “guide points” provided by the analyst, as well as computer-generated data points calculated from the image using an edge detection algorithm. Automatic feature tracking was used to track points throughout the cardiac cycle using non-rigid registration in both short and long axis images [12]. Information from all slices and frames was integrated into the time-varying 3D model to provide a 3D representation for the beating heart surfaces (endocardium and epicardium). The model was registered to each case using fiducial landmarks which were manually defined at the hinge points of the mitral valve on the long axis images, and at the insertions of the right ventricular free wall into the inter-ventricular septum. These were used to define a standard coordinate system which mapped the position of the model shape parameters to consistent positions registered to the anatomy of each heart. This method has been previously validated against autopsy LV mass, in patients against manually drawn contours and in healthy volunteers against flow-derived measurements of cardiac output [11].

**Figure 2:**
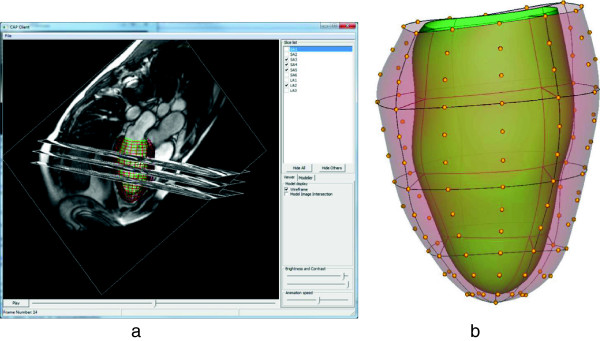
**Mathematical shape model. a)** 3D display of short and long axis images with the model embedded (CAP visualization tool available from http://www.cardiacatlas.org). LV shape model shown as wire-frame. **b)** Finite element description of left ventricular shape. Model parameters are shown as yellow points (endocardial parameters not shown). Element boundaries shown as lines; endocardial surface shaded green; epicardial surface shaded red and transparent.

The finite element coordinates were used to provide the atlas coordinates of the LV: each point is assumed to be in approximately in the same anatomical location in every heart [13]. Shape parameters of the atlas were provided by the finite element control points for each of the epicardial and endocardial surfaces. These points were evenly spaced around the heart and intuitively control the position of the model locally at each point. Figure 2 shows the spatial distribution of the shape parameters. This parametric representation for the heart also enabled the surfaces to be sampled at arbitrary resolution anywhere in the heart (in this paper we used 1,089 points for each surface) for statistical shape comparisons.

### Shape correction

**Figure 3:**
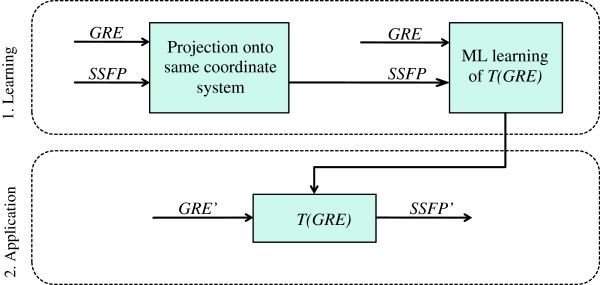
**Signal model framework for learning and application.** The transformation *T(GRE)* was learned from paired sets of N models with both GRE and SSFP versions, using maximum likelihood (ML). The transformation was then applied to separate datasets *GRE’* to produce SSFP estimates *SSFP’*.

The bias correction process involved two steps (Figure 3): a learning step in which the transformation was generated, and an application step in which it was applied.

Firstly, the SSFP finite element model for each volunteer was re-parameterized using the same coordinate system as the GRE model (Figure 3). This step was necessary since the transformation was designed to be applied to the GRE parameterization. Secondly, for each shape parameter, the mean and standard deviation of the distribution of locations were calculated with respect to the GRE cases and the SSFP cases.

**Figure 4:**
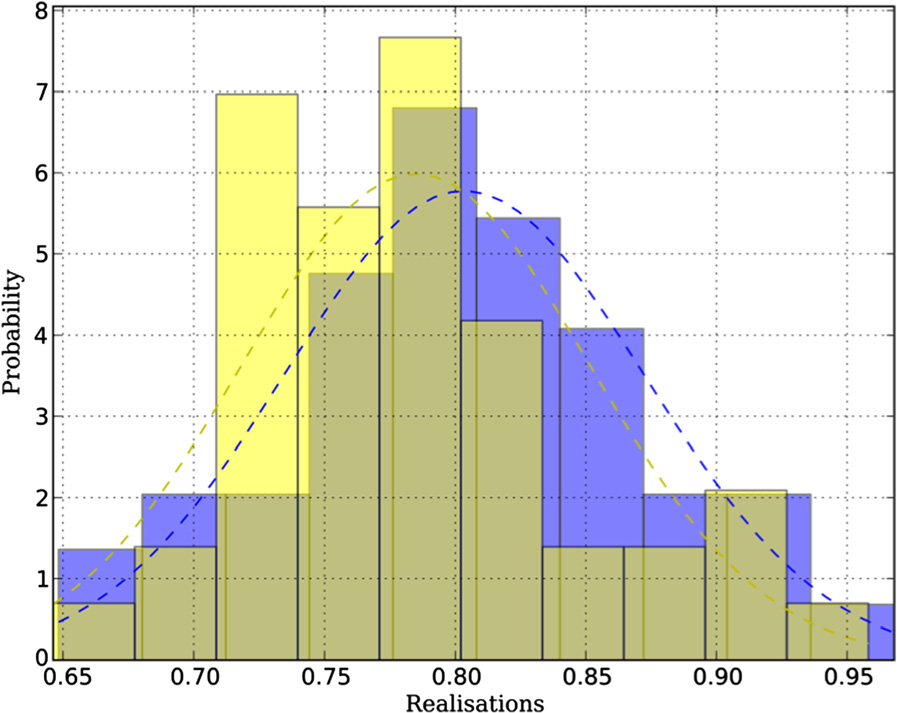
**Sample distribution of a single model parameter for N = 46 cases.** The blue distribution corresponds to the GRE protocol and the yellow to SSFP. Both passed Anderson’s test of normality with an α-level of 5%. Maximum likelihood Gaussian distributions are also shown.

We assumed that the parameters would be normally distributed due to the central limit theorem, since they arise from the combination of a number of processes (including age, gender, image formation, analysis). A test for normality confirmed that the parameter distributions could be approximated by Gaussians; of all the parameter distributions, 88% passed Anderson’s test of normality [14], which indicates that they were likely to have been derived from normal processes (with an α-level of 5%). The parameters of the Gaussian distributions were estimated by means of maximum likelihood [15]. Examples of these distributions are shown in Figure 4.

For each shape parameter, the transformation *T(GRE)* was estimated from the GRE and SSFP training models (N = 46) as shown in Figure 3. The transformation could be then applied to a separate set of GRE datasets *GRE’* to provide bias-corrected SSFP datasets *SSFP’* (Figure 3). The transformation *T(GRE)* was defined for each shape parameter by a z-score correction between the two populations:

(1) SSFP'≡TGRE'=GRE'−μGREσGREσSSFP+μSSFP

Given the four Gaussian-distribution parameters estimated by maximum likelihood (mean and standard deviation of the GRE and SSFP training sets, namely (*μ*_*GRE*_, *σ*_*GRE*_) and (*μ*_*SSFP*_, *σ*_*SSFP*_) respectively) we can estimate any SSFP value from its corresponding GRE value using Eqn 1.

A variety of different types of transformation were investigated and the results are summarized in the Appendix. Eqn 1 was the only transform which minimized both the residual surface and volume bias of the models in our experiments.

### Validation

Validation was performed by means of leave-one-out experiments, in which the transformation was trained using N = 45 cases, and errors in surface position and volume calculated for the remaining case. This process was repeated 46 times, leaving each case out in turn, and the resulting errors averaged.

Error analysis was performed in the leave-one-out experiments to examine both global volume errors and local surface bias errors. Firstly, we estimated the mass and volume of the transformed LV models, in comparison with the original SSFP mass and volumes, in order to confirm that the local correction of shape parameters also corrected global clinical indices of mass and volume [10]. Secondly, the local error in each surface position was estimated by computing the residual bias between corresponding points in the estimated and measured SSFP surface, using a surface sampling of 1,089 points.

### Application

To evaluate the effects of shape bias removal in a typical application, we compared 300 cases from MESA with 105 cases from DETERMINE obtained from the CAP database. All cases were de-identified and contributed to the CAP database with approval from the local Institutional Review Boards. For the DETERMINE cases, two expert observers visually scored areas of late gadolinium enhancement (LGE) by consensus [16], on each of the 17 AHA regions [17]. LGE scores were categorized into 5 grades (0–4) according to the transmural thickness of enhancement on the LGE scan: 0% (0), 1–25% (1), 26–50% (2), 51–75% (3) and 76–100% (4) of the wall thickness. The multivariate Hotelling’s *T*^*2*^ test (assuming unequal variance) was used to test for regional shape differences at end-diastole (ED) between DETERMINE segments with an LGE score of 2 or higher and the corresponding segments from the 300 MESA controls (which were assigned a label of 0). These differences were quantified with and without the correction of shape bias from GRE to SSFP in the MESA control cases. In addition, average shape differences were visualized between a subcohort of DETERMINE patients with infero-lateral infarction (N = 27) and the 300 MESA cases using the Hotelling’s *T*^*2*^ test (the extension of the standard t-test to multiple dimensions) on a point by point basis.

### Statistics

Different regression models were examined using R v. 2.11 (R Development Core Team, 2011) to find a mapping which minimized both the shape and volume bias (see Appendix).

The Hotelling’s *T*^*2*^ statistic was used to evaluate shape changes in each AHA segment between cohorts [18].

## Results

### Shape and volume bias

**Figure 5:**
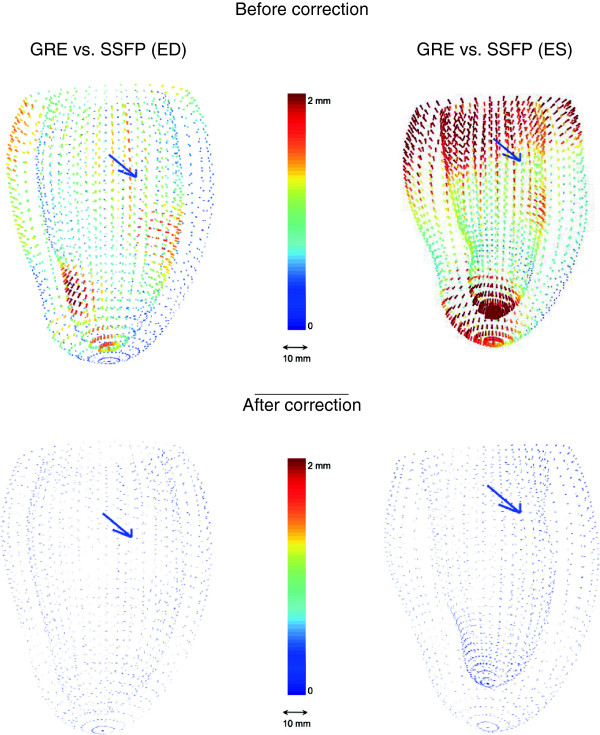
**Average difference due to protocol bias in the LV at ED (left) and ES (right).** The top two figures show the bias before correction and the bottom ones afterwards. The arrow points from the center of the blood pool toward the septum.

Figure 5 shows the average differences between GRE and SSFP at ED and ES for the 46 volunteers. The most significant regional differences appear in the apical endocardium and around the papillary muscles. Also some differences arise near the basal plane, especially at ES. These differences are physically reasonable since GRE contrast is dependent on blood flow to a greater extent than SSFP, leading to possible regional differences where apical trabeculation or papillary muscles disrupt the local blood flow [10]. Similarly, the basal differences are likely due to differences in the appearance of the long axis images around the mitral valve (see Figure 1 for image examples). Table 1 reports the mean and standard deviation of the computed volumes for these experiments. Left-ventricular mass (LVM) was calculated at ED as the myocardial volume times 1.05 g/ml.

**Table 1:** Protocol bias

	**EDV (ml)**	**ESV (ml)**	**LVM (g)**
GRE	126.2 ± 27.0	52.8 ± 12.6	145.2 ± 33.2
SSFP	134.1 ± 28.3	52.8 ± 13.7	131.1 ± 31.6
Estimated SSFP	133.7 ± 29.6	53.2 ± 14.4	130.1 ± 30.9
Volume error pre correction	−7.9 ± 12.3	0.0 ± 8.2	14.1 ± 10.5
Volume error post correction	−0.4 ± 14.6	0.4 ± 10.4	−1.1 ± 10.8
	**ED RMS (mm)**	**ES RMS (mm)**	
Surface bias pre correction	0.75	1.39	
Surface bias post correction	0.06	0.07	

Cavity volume at end diastole (EDV), end systole (ESV), myocardium mass (LVM) and leave-one-out errors in volume and surface root mean squared error (RMS) for the indicated sets (N = 46).

The Table 1 volume errors show that the leave-one-out corrected volumes agree with the measured SSFP volumes with an absolute average bias of ≤ 1 ml. Table 1 also shows that the local surface biases were also greatly reduced.

### Visualization of regional shape abnormalities

The transformation from GRE to SSFP shape models was applied to 300 asymptomatic volunteers from the MESA (GRE) study in order to make direct comparisons with 105 patients with myocardial infarction from the DETERMINE (SSFP) study.

**Figure 6:**
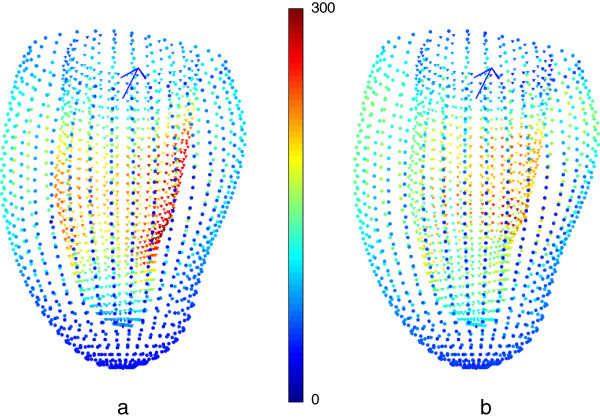
**Application of shape correction to the analysis of myocardial infarction. a)** Hotelling’s *T*^*2*^ for the infero-lateral DETERMINE subcohort (N = 27) compared to the MESA cases (N = 300) without any protocol-bias correction. **b)** Hotelling’s *T*^*2*^ statistic after the protocol-bias correction. Red shows most significant differences while blue represents least significant differences. The papillary muscle significant difference due to protocol bias disappears after correction. Viewpoint is from the lateral wall: the arrow points from the center of the blood pool toward the septum.

Figure 6 visualizes the shape differences at ED with and without the bias correction by means of Hotelling’s *T^2* statistic (9) in those DETERMINE cases with infero-lateral infarction (N = 27). This test compares mean and variance of the surface points in a multi-dimensional extension of the Student’s t-test. If the bias is not corrected, differences in shape around the papillary muscles (both anterior and inferior) appear to show differences due to infarction, whereas after correction this apparent abnormality is removed.

### Quantification of regional shape differences in LGE

Regional differences in shape were examined using the AHA 17 segment model. Each finite element model was sampled uniformly by 200 points in each AHA segment. Hotelling’s *T*^*2*^ was then applied on a regional basis to test for statistically significant shape changes in those segments with >50% transmural LGE score.

**Figure 7:**
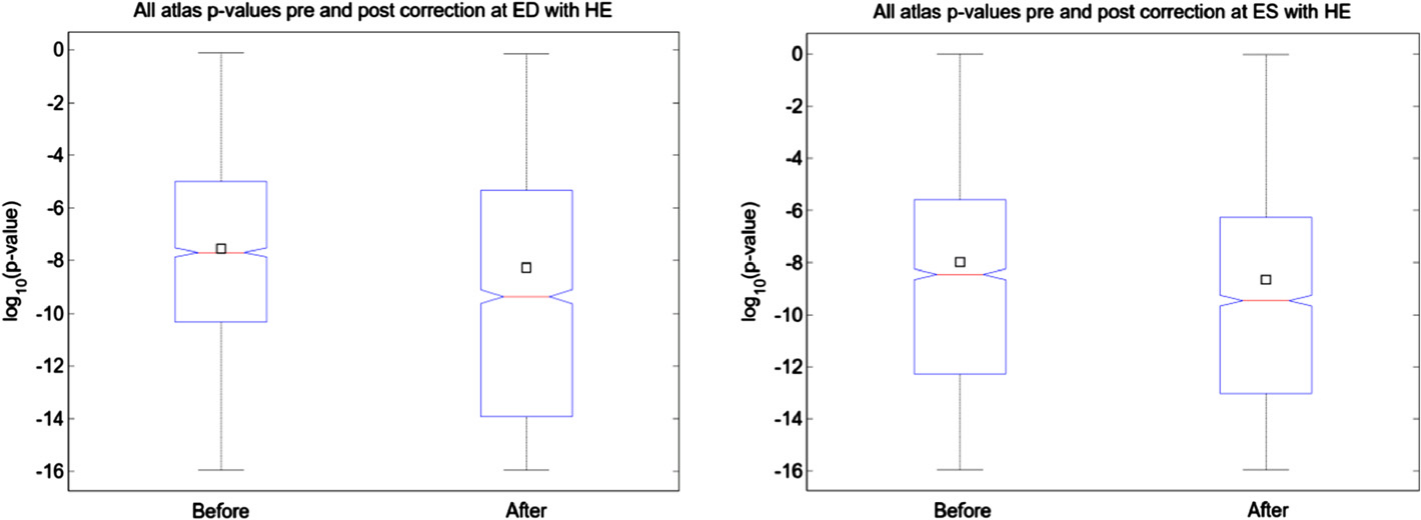
**Distribution of Hotelling’s p-values between DETERMINE segments with >50% transmural LGE, and MESA cases for 2,500 evenly-sampled points (in logarithmic scale).** The box-plots show how, at both ED and ES, the differences become more significant after correction. The 25th percentile, the median and the 75th percentile are drawn as boxes and the mean is represented by a square.

Figure 7 shows that after correction, the significance of the separation of the two populations tended to be greater, i.e. the distribution of p-values of the post-transformed population for abnormal cases was more significant, on average by a power of ten, both at ED and ES. This implies that, although the point distributions have had some differences removed by the bias correction, the statistical separation between patient cohorts was actually improved. This result shows that bias correction can improve the statistical significance of regional shape differences between cohorts using an atlas-based analysis.

## Discussion and conclusions

Atlas-based analysis of heart shape and function shows promise in providing new clinical information about the degree and progression of heart disease [6,7]. The data and software components used in the Cardiac Atlas Project, including the database infrastructure and visualization tool, are open-source and available for download at the website (http://www.cardiacatlas.org). Atlas-based methods establish a common coordinate system [19] which can be used to describe statistical shape changes. These methods create a template of the anatomy which is then warped to each case [20-22]. In this study we have used a finite element computational model of the left ventricle which was mapped to the anatomy of each case [12]. A similar map was used to guide biopsy samples with an accuracy of 0.3 ± 3.7 mm [13]. However, bias arising from different image protocols will lead to systematic bias in the shape parameters which will confound analysis of pathological variation. To our knowledge, this is the first attempt at mapping and correcting the protocol effect across a population of shape models at the local parameter level. The bias between GRE and SSFP imaging protocol was found to be regional in nature, associated with regional differences in flow enhancement. A z-score correction method was used to transform regional shape parameters. This transformation was the only one examined which corrected both local surface bias and global bias in mass and volume. The bias correction enabled visualization of regional wall shape abnormalities due to myocardial infarction to be corrected for imaging protocol on a regional basis. Despite the reduction of false positives, the method enabled better characterization of the segmental shape differences between segments with and without scar, as defined by >50% transmural extent by LGE. Note that this method corrects average error across all cases (at the population level) —rather than absolute error for each model— since the mapping is designed to correct statistical bias only. The method does not reduce the variation of shape present in a population.

**Figure 8:**
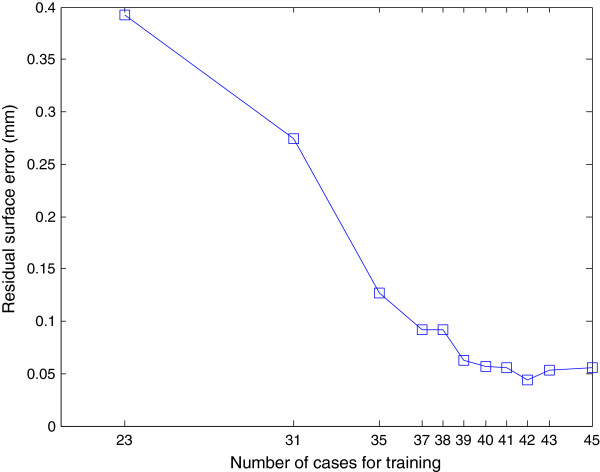
Residual surface error (mm) for different number of cases in the training dataset.

In order to determine how many cases are required in the training set to determine the transformation, we performed additional validation experiments by leaving out more cases in the training step. Figure 8 shows the results of these experiments, showing that 39 cases are sufficient for stable and robust estimation of the shape bias correction transform in this application, with an average residual surface error of less than 0.1 mm. To quantify the effect of inter-observer variation on the bias transformation, the shape bias was recalculated using guide-point models obtained by another observer (both SSFP and GRE). Fewer than 0.01% of points reported significant differences from the first analysis (p < 0.05). This suggests that the regional differences are robust to observer variation in the placement of surface boundaries on the images.

Limitations of this study include the use of healthy volunteers to train the transformation, thus limiting the transformation to relatively normal heart shapes. Since we applied the transformation only to asymptomatic volunteers from MESA, we do not expect a significant error due to this effect in the current application. However, it is not known whether the transformation derived from this dataset will show the same degree of robustness when applied to patients with disease, such as hypertensive hypertrophy where the wall becomes greatly thickened, or heart failure where slow moving blood with GRE imaging in poor heart function may accentuate differences. Age may also affect the transformation, but we expect these effects to be much less than the effect of imaging protocol. Another limitation is the requirement for a training group examined with both imaging protocols. Further work is required to develop transformations without the need for such a training set. This might involve simulating images using different protocols [23].

Our method is not limited to the application described here (SSFP and GRE), and can be applied in a variety of different applications. For example, inter-observer bias arises when images are analyzed by different readers who have different interpretations of contour location. Also different clinical studies use core laboratories which vary in analysis protocol and software. If Analysis A and Analysis B use different readers/protocols/software, this bias must be corrected before results can be pooled in any meta-analysis. By analyzing a subset of cases from both A and B with Analysis C, the transforms can be learned to map A to C and B to C. All studies can then be mapped to a common standard, thereby removing this source of bias. In addition, this method can be used to compare results from different imaging modalities, e.g. CT vs CMR.

## Appendix

### Comparison of transformations

A number of different transformation methods were examined. These included (parameter estimation represented as *ŷ = f(x)*, where *x* refers to GRE location and *ŷ* to the estimated SSFP location):

1. Intercept only (zero slope): *ŷ = c*, where *c* is a constant estimated separately for each parameter.

2. Slope and intercept: *ŷ = m x + c* where *m* and *c* are constants estimated separately for each parameter.

3. Slope only (zero intercept): *ŷ = m x*, where *m* is a constant estimated separately for each parameter.

4. Forced identity slope and varying intercept: *ŷ = x + c* where *c* is a constant estimated separately for each parameter.

5. Maximum likelihood estimation (z-score correction) as defined in Equation 1.

6. Multivariate approach with grouped parameters: *ŷ = Ax + C* where *A* is a 4 × 4 constant matrix and *C* is a four element constant vector, estimated for each group of 4 shape parameters around each node of the finite element model. This enables modeling of covariances around each node.

7. Pseudo-inverse global transformation matrix: *ŷ = Px* where *P* is the pseudo-inverse of the data matrix from set *X*. In theory this enables covariances between all shape parameters.

All these models can be interpreted as different design matrices. The first four are the classic univariate (one transform per parameter, with each parameter treated independently) linear models. Case 5 is also univariate. In case 6, parameters were grouped by finite-element nodes, four parameters per node, which were related between models by a 4 × 4 matrix. In case 7, the design matrix was computed by use of the pseudo-inverse (*X*^*+*^) matrix of the data matrix *X* from set *X*. Note that in this last approach, the number of coefficients is larger than the available data points (215 vs. 46) hence the mapping matrix is unstructured and noisy; however, we still include it for comparison purposes.

As can be seen in Table 2, case 5 reported the best results and it was therefore chosen as the reference regression model applied in all further work.

**Table 2:** Surface and volume errors for the regression models

**Model**	**ED**	**ES**	**LVM**
Intercept only			
Estimated SSFP	130.7 ± 33.9 ml	49.2 ± 12.7 ml	132.8 ± 34.4 g
Residual volume error	−3.4 ± 26.3 ml	−3.7 ± 11.3 ml	1.7 ± 26.5 g
Residual surface error	0.14 mm	0.26 mm	
Slope and intercept			
Estimated SSFP	130.3 ± 27.1 ml	49.3 ± 11.0 ml	131.3 ± 29.6 g
Residual volume error	−3.8 ± 13.2 ml	−3.6 ± 8.0 ml	0.1 ± 12.2 g
Residual surface error	0.10 mm	0.20 mm	
Slope and forced origin			
Estimated SSFP	130.5 ± 27.7 ml	49.2 ± 11.5 ml	131.1 ± 30.3 g
Residual volume error	−3.7 ± 12.6 ml	−3.7 ± 8.2 ml	0.0 ± 10.3 g
Residual surface error	0.13 mm	0.27 mm	
Forced identity and intercept			
Estimated SSFP	132.0 ± 28.0 ml	50.6 ± 11.9 ml	131.5 ± 30.3 g
Residual volume error	−2.1 ± 12.6 ml	−2.2 ± 8.2 ml	0.3 ± 10.3 g
Residual surface error	0.08 mm	0.16 mm	
**Maximum likelihood estimated**			
**Estimated SSFP**	**133.7 ± 29.6 ml**	**53.2 ± 14.4 ml**	**130.1 ± 30.9 g**
**Residual volume error**	**−0.4 ± 14.6 ml**	**0.4 ± 10.4 ml**	**−1.1 ± 10.8 g**
**Residual surface error**	**0.06 mm**	**0.07 mm**	
Nodal-grouped parameters			
Estimated SSFP	130.5 ± 28.0 ml	49.5 ± 11.7 ml	130.2 ± 27.4 g
Residual volume error	−3.6 ± 12.6 ml	−3.3 ± 7.7 ml	−0.9 ± 13.2 g
Residual surface error	0.09 mm	0.21 mm	
Pseudo-inverse global			
Estimated SSFP	137.9 ± 33.1 ml	54.4 ± 15.3 ml	130.1 ± 32.7 g
Residual volume error	3.8 ± 16.3 ml	1.6 ± 12.7 ml	−1.2 ± 19.0 g
Residual surface error	0.28 mm	0.34 mm	

For original GRE and SSFP volumes see Table 1.

Table 2 shows the results of the various mapping methods tested in terms of volume, mass and surface error. As expected, all methods reduced the surface bias to some degree and all surface errors are low in terms of clinical requirements. However, the MLE method was best in terms of both residual surface bias and mass and volume estimates.

## Competing interests

The authors declare that they have no competing interests.

## Authors’ contributions

PM-G drafted the manuscript and participated in the study design, data analysis, data interpretation and manuscript editing. BRC participated in the study design, data interpretation and manuscript editing. DAB participated in the study design, data acquisition and manuscript editing. JPF participated in the study design, data interpretation and manuscript editing. AHK participated in the study design, data acquisition and manuscript editing. DCL participated in the study design, data acquisition and manuscript editing. JACL participated in the study concepts, data acquisition and manuscript editing. AS participated in the, study design, data analysis and manuscript editing. AAY conceived the study and participated in the study design, data interpretation and manuscript editing. All authors read and approved the final manuscript.
